# Minimally Invasive Revisional Bariatric Surgery in a MBSAQIP Accredited High-Volume Center

**DOI:** 10.3389/fsurg.2022.880044

**Published:** 2022-04-01

**Authors:** Julia Xie, Nicolas H. Dreifuss, Francisco Schlottmann, Antonio Cubisino, Alberto Mangano, Carolina Vanetta, Carolina Baz, Valentina Valle, Francesco M. Bianco, Antonio Gangemi, Mario A. Masrur

**Affiliations:** Division of General, Minimally Invasive, and Robotic Surgery, Department of Surgery, University of Illinois at Chicago, Chicago, IL, United States

**Keywords:** robotics, bariatric surgery, minimally invasive, sleeve gastrectomy, gastric bypass

## Abstract

**Background:**

With the rising number of bariatric surgeries performed annually, there has also been an increase in revisional bariatric surgeries (RBS). The aim of this study is to evaluate the safety and postoperative outcomes of RBS performed with a minimally invasive approach.

**Methods:**

Retrospective analysis on a prospectively collected database of patients who underwent minimally invasive RBS between 2012 and 2019. Primary endpoints were conversion rate, major morbidity, mortality, and 30-day reoperation rate. Comparative analysis of laparoscopic adjustable gastric banding (LAGB) conversion to sleeve gastrectomy (SG) vs. conversion to Roux-en-Y gastric bypass (RYGB) was performed.

**Results:**

A total of 221 patients underwent minimally invasive RBS, 137 (62%) laparoscopically and 84 (38%) robotically. The most common RBS were LAGB to SG (59.3%) and LAGB to RYGB conversions (16.7%). The main indication was weight loss failure (88.7%). Conversion rate, major morbidity, and mortality were 0.9, 3.2, and 0.4%, respectively. Urgent reoperation was required in 3.2% of cases. Total weight loss at 1 and 2-years follow- were 14.3 and 17.3%, respectively. Comparative analysis of LAGB conversion to SG vs. RYGB showed similar major morbidity (SG: 2.3% vs. RYGB 0%, *p* = 1). Greater total weight loss was achieved in LAGB to RYGB conversions at 1-year (SG: 14.8% vs. RYGB 25.3%, *p* < 0.001).

**Conclusions:**

Minimally invasive RBS can be performed safely in a broad patient population with low conversion and complication rates, and improved weight loss outcomes. LAGB to RYGB conversions are associated with greater weight loss. Further randomized trials are needed to draw more conclusive recommendations.

## Introduction

Bariatric surgery has been shown to be the most effective treatment to achieve sustained weight loss and to improve obesity-related comorbidities (1, 2). Revisional bariatric surgery (RBS) is indicated when primary procedures fail to produce adequate weight loss or result in complications (3). They most commonly follow laparoscopic adjustable gastric banding (LAGB) (4). Compared to the index procedure, higher complication rates are observed in revisional cases (3).

Similar to the results seen in primary bariatric operations, minimally invasive techniques are also associated with lower complication rates in revisional procedures when compared to the conventional approach (5, 6). In recent years, the utilization of the robotic platform significantly increased across specialties due to improved surgical ergonomics, 3-D magnification, elimination of physiologic tremor, and seven degrees of freedom of the instruments with improved dexterity. However, concerns regarding the increased cost and longer operative time limited its widespread adoption in bariatric surgery. Although there is conflicting data in literature, the robotic approach for RBS seems to have a similar safety profile when compared to the laparoscopic approach (7–11).

LAGB was first introduced in 1993 and became the most popular bariatric procedure performed in the early 2000s (12, 13). However, an increasing number of these patients needs RBS due to poor long-term weight loss outcomes and complications. The removal rate increases by 3–4% each year, with the majority of patients requiring revisional surgery long-term (14). After LAGB removal, most patients undergo conversion to sleeve gastrectomy (SG) or Roux-en-Y gastric bypass (RYGB) (4). Currently, there is contradictory data from limited studies comparing the outcomes in LAGB conversion to SG and conversion to RYGB (15–17). The aim of this study is to evaluate the applications, safety and postoperative outcomes of RBS performed minimally invasively, including a comparative analysis of LAGB conversion to SG vs. conversion to RYGB.

## Materials and Methods

### Study Design

A retrospective analysis was performed on a prospectively collected database of patients who underwent minimally invasive RBS between 2012 and 2019. Revisional procedures were all performed laparoscopically or with the DaVinci Surgical System (Intuitive Surgical Inc., Sunnyvale, CA, USA) by 5 bariatric surgeons at our institution experienced in primary and RBS as well as laparoscopic and robotic approaches. Index procedures included LAGB, vertical banded gastroplasty (VBG), gastric plication (GP), SG, RYGB, and biliopancreatic diversion with duodenal switch (BPD/DS). Indications for revision were weight loss failure (WLF), severe gastroesophageal reflux disease (GERD), LAGB erosion or acute slippage, gastric stenosis, gastro-gastric fistula, dumping syndrome, and malnutrition. LAGB removals alone (without subsequent conversion) and candy cane resections were not considered RBS and were excluded from the analysis. WLF was defined per Reinhold criteria as insufficient weight loss or weight regain after bariatric procedure (18). GERD was defined by the presence of symptoms or esophagitis on esophagogastroduodenoscopy (EGD) despite medical therapy. All patients underwent a complete medical, nutritional and psychological evaluation prior to the revisional procedure. Upper gastrointestinal series and/or endoscopy were also performed preoperatively Manometry, pH monitoring, computed tomography scan, and gastric emptying studies were performed if clinically indicated. Postoperative follow-up in clinic was at 2 weeks, 1 month, every 3 months for the first 2 years, and then annually. The study was approved by the Institutional Review Board (IRB) of our hospital.

### Data Items

Preoperative variables collected include age, sex, comorbidities, American Society of Anesthesiologists (ASA) classification, weight and body mass index (BMI) at revision, type of primary and revisional bariatric operations performed, time interval between them, indication for revision, and number of revisions per patient. Operative variables collected include surgical approach (laparoscopic or robotic assisted), associated procedures, operative time, conversion rate, estimated blood loss (EBL), and intraoperative complications. Postoperative variables collected include intensive care unit (ICU) admission, length of stay (LOS), postoperative complications (according to the Clavien-Dindo classification), major morbidity (Clavien-Dindo ≥IIIa), mortality, 30-day readmission, and 30-day reoperation rates. Long-term complications requiring surgical intervention and weight loss outcomes at follow-up (3, 6, 12, 18, 24, 36, 48, 60 months), measured as change in BMI, excess weight loss (%EWL), and total weight loss (%TWL), were also recorded.

The primary outcomes of interest were: conversion rate, major morbidity, mortality, and 30-day reoperation rate. Secondary outcomes of interest include: operative time, LOS, long-term complications requiring surgical intervention, and weight loss. A comparative analysis of postoperative and weight loss outcomes in LAGB to SG vs. LAGB to RYGB was also performed.

### Statistical Analysis

Utilizing R Project for Statistical Computing, *t*-test was used for continuous variables, and Fisher's exact test was used for categorical variables. A significance threshold of α = 0.05 was decided a priori to determine statistical significance.

## Results

**Figure 1:**
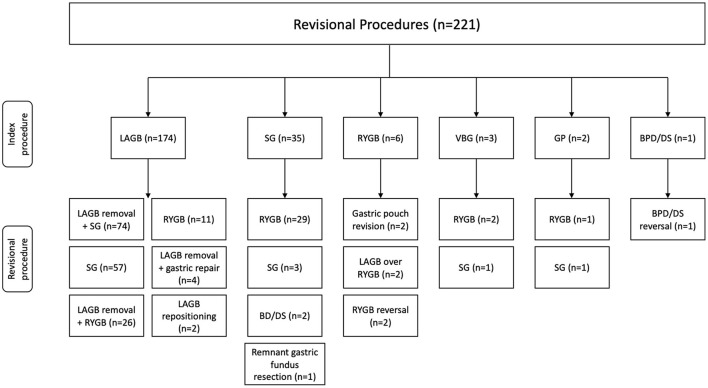
Index and revisional procedures. LAGB, laparoscopic adjustable gastric banding; SG, sleeve gastrectomy; RYGB, Roux-en-Y gastric bypass gastric banding; VBG, vertical banded gastroplasty; GP, gastric plication; BPD/DS, biliopancreatic diversion with duodenal switch.

A total of 221 revisional bariatric procedures were performed utilizing a minimally invasive approach. The mean age of the study population was 44.3 (20–80) years with the majority being female (94.1%). The most common comorbidities were hypertension 39.8%, diabetes mellitus 26.2%, asthma 21.7%, and GERD 19.5%. The majority of patients were ASA Class III (75.6%). Mean weight and BMI at revision were 124.2 (54–226) kg and 45.6 (18–91) kg/m^2^, respectively. The average interval between index and revisional procedures was 88.3 (9–588) months and the average number of revisions per patient was 1.5 (1–5) (Table 1). The index and corresponding revisional procedures are outlined in Figure 1. The three most common indications for revision were WLF (88.7%), GERD (5.4%), and LAGB erosion (1.8%) (Table 2).

**Table 1:** Preoperative variables.

Age, years, mean (range)	44.3 (20–80)
**Sex, *n* (%)**	
Female	208 (94.1%)
Male	13 (5.9%)
Weight at revision, kg, mean (range)	124.2 (54–226)
BMI at revision, kg/m^2^, mean (range)	45.6 (18–91)
**Comorbidities, *n* (%)**	
HTN	88 (39.8%)
DM	58 (26.2%)
Asthma	48 (21.7%)
GERD	43 (19.5%)
OSA	38 (17.2%)
HLD	37 (16.7%)
Smoker	13 (5.9%)
COPD	4 (1.8%)
**ASA, *n* (%)**	
I	23 (10.4%)
II	31 (14%)
III	167 (75.6%)
Interval between procedures, months, mean (range)	88.3 (9–588)
Number of revisions, mean (range)	1.5 (1–5)

*BMI, body mass index; COPD, chronic obstructive pulmonary disease; DM, diabetes mellitus; HTN, hypertension; HLD, hyperlipidemia; OSA, obstructive sleep apnea; GERD, gastroesophageal reflux disease; ASA, American Society of Anesthesiologists*.

**Table 2:** Indication for RBS.

**Index Procedure**	**Revisional Procedure**	**Indication**
LAGB (*n =* 174)	LAGB removal + SG (*n =* 74)	WLF (*n =* 70) GERD (*n =* 3) LAGB acute slippage (*n =* 1)
	SG (*n =* 57)	WLF (*n =* 57)
	LAGB removal + RYGB (*n =* 26)	WLF (*n =* 25) GERD (*n =* 1)
	RYGB (*n =* 11)	WLF (*n =* 9) GERD (*n =* 1) LAGB acute slippage (*n =* 1)
	LAGB removal + gastric repair (*n =* 4)	LAGB erosion (*n =* 4)
	LAGB repositioning (*n =* 2)	GERD (*n =* 1) LAGB acute slippage (*n =* 1)
SG (*n =* 35)	RYGB (*n =* 29)	WLF (*n =* 22) GERD (*n =* 6) Gastric stenosis (*n =* 1)
	SG (*n =* 3)	WLF (*n =* 3)
	BPD/DS (*n =* 2)	WLF (*n =* 2)
	Remnant gastric fundus resection (*n =* 1)	WLF (*n =* 1)
RYGB (*n =* 6)	Gastric pouch revision (*n =* 2)	Gastrogastric fistula (*n =* 2)
	LAGB over RYGB (*n =* 2)	WLF (*n =* 2)
	RYGB reversal (*n =* 2)	Dumping syndrome (*n =* 1) Malnutrition (*n =* 1)
VBG (*n =* 3)	RYGB (*n =* 2)	WLF (*n =* 2)
	SG (*n =* 1)	WLF (*n =* 1)
GP (*n =* 2)	RYGB (*n =* 1)	WLF (*n =* 1)
	SG (*n =* 1)	WLF (*n =* 1)
BPD/DS (*n =* 1)	BPD/DS reversal (*n =* 1)	Malnutrition (*n =* 1)

*LAGB, laparoscopic adjustable gastric banding; SG, sleeve gastrectomy; RYGB, Roux-en-Y gastric bypass; VBG, vertical banded gastroplasty; GP, gastric plication; BPD/DS, biliopancreatic diversion with duodenal switch; WLF, weight loss failure; GERD, gastroesophageal reflux disease*.

Surgical approach was laparoscopic in 137 (62%) cases and robotic in 84 (38%) cases. An associated procedure was performed in 37 (16.7%) of RBS and included: 32 (14.5%) hiatal hernia repairs, 2 (0.9%) cholecystectomies, 2 (0.9%) ventral hernia repairs, and 1 (0.4%) incisional and hiatal hernia repair. The mean operative time was 149.2 (45–391) min and mean EBL was 25.7 (5–800) mL. One (0.4%) intraoperative complication (splenic vessel injury) and 2 (0.9%) conversions from laparoscopic to open approach (splenic vessel injury and extensive adhesions) were recorded.

Four patients (1.8%) required immediate postoperative ICU admission. Two patients had extensive cardiovascular histories, one had a splenic vessel injury with significant blood loss, and another patient had a severe penicillin allergy requiring antibiotic treatment for an abdominal wound infection. Major morbidity was 3.2% (7 patients). One patient underwent percutaneous drainage of an abdominal collection due to a staple line leak. Seven patients (3.2%) underwent urgent reoperation for abdominal abscess, incarcerated incisional hernia, small bowel obstruction, staple line leak, perforated remnant stomach after redo gastrojejunostomy of RYGB, postoperative hemorrhage, and severe ileus. Mortality was 0.4%; 1 patient died of septic shock after LAGB removal and conversion to RYGB complicated by severe ileus and intestinal perforation. The mean LOS was 2 (0–27) days. Thirteen patients (5.9%) were readmitted (Table 3). Similar conversion rates (laparoscopic: 1.5% vs. robotic: 0%, *p* = 0.53), major morbidity (laparoscopic: 3.6% vs. robotic 2.4%, *p* = 0.71), mortality (laparoscopic: 0% vs. robotic: 1.2%, *p* = 0.38), and reoperation rates (laparoscopic: 2.9% vs. robotic: 3.6%, *p* = 1) were found in robotic and laparoscopic revisional cases.

**Table 3:** Postoperative variables.

Postoperative ICU admission, *n* (%)	4 (1.8%)
LOS, days, mean (range)	2 (0–27)
**Clavien-Dindo classification, *n* (%)**	
I	15 (6.8%)
II	2 (0.9%)
IIIa	1 (0.4%) Staple line leak
IIIb	4 (1.8%) Abdominal abscess Incarcerated incisional hernia Staple line leak Small bowel obstruction
IV	2 (0.9%) Perforated gastric ulcer Pulmonary embolism
V	1 (0.4%) Septic shock
Overall morbidity, *n* (%)	25 (11.3%)
Major morbidity, *n* (%)	7 (3.2%)
Readmission within 30 days, *n* (%)	13 (5.9%)
Reoperation within 30 days, *n* (%)	7 (3.2%)
**Long-term complications: *n* (%)**	
Ventral hernia	7 (3.2%)
Petersen hernia	2 (0.9%)
Small bowel obstruction	2 (0.9%)

*ICU, intensive care unit; LOS, length of stay*.

The mean follow-up was 21 (1–91) months. Long-term complications requiring surgical intervention included 7 (3.2%) ventral hernias, 2 (0.9%) Petersen hernias, and 2 (0.9%) small bowel obstructions. At 1-year follow-up (107 seen/220 available patients), change in BMI was −6.9 kg/m^2^, %EWL 31.2%, and %TWL 14.3%. At 2-years follow-up (70 seen/220 available patients), change in BMI was −7.6 kg/m^2^, %EWL 33.8%, and %TWL 17.3%.

LAGB removal and conversion to SG and RYGB was performed in 131 (56.5% one stage) and 37 patients (70.3% one stage), respectively. Overall morbidity was similar for one stage and two stage LAGB to RYGB or SG conversions (One stage: 6% vs. two stage: 4.4%, *p* = 0.65). Patients who underwent LAGB to SG conversions were older (SG: 44.8 years vs. RYGB: 40.4 years, *p* = 0.003), and a greater percentage were ASA Class III (SG: 85.5% vs. RYGB: 51.4%, *p* < 0.001). The weight (SG: 125.6 kg vs. RYGB: 124.9 kg, *p* = 0.85) and BMI (SG: 46.3 vs. RYGB: 47.5 kg/m^2^, *p* = 0.38) at revision were similar in both groups. Postoperative complications (SG: 3.8% vs. RYGB: 10.8%, *p* = 0.11), major morbidity (SG: 2.3% vs. RYGB: 0%, *p* = 1), mortality (SG: 0% vs. RYGB: 2.7%, *p* = 0.22), 30-day readmission (SG: 2.3% vs. RYGB: 0%, *p* = 1), 30-day reoperation (SG: 1.5% vs. RYGB: 2.7%, *p* = 0.53), and long-term complications (SG: 0.8% vs. RYGB: 5.4%, *p* = 0.12) were comparable between the two groups (Table 4).

**Table 4:** Postoperative outcomes in LAGB to SG vs. LAGB to RYGB.

	**LAGB to SG (*n =* 131)**	**LAGB to RYGB (*n =* 37)**	***p*-value**
**Clavien-Dindo classification**, ***n*** **(%)**			
I	2 (1.5%)	3 (8.1%)	0.07
II	0 (0%)	0 (0%)	1
IIIa	1 (0.8%) Staple line leak	0 (0%)	1
IIIb	1 (0.8%) Incarcerated incisional hernia	0 (0%)	1
IV	1 (0.8%) Pulmonary embolism	0 (0%)	1
V	0 (0%)	1 (2.7%) Septic shock	0.22
Overall morbidity, *n* (%)	5 (3.8%)	4 (10.8%)	0.11
Major morbidity, *n* (%)	3 (2.3%)	0 (0%)	1
Readmission, *n* (%)	3 (2.3%)	0 (0%)	1
Reoperation, *n* (%)	2 (1.5%)	1 (2.7%)	0.53
Long-term complications, *n* (%):	1 (0.8%)	2 (5.4%)	0.12

*LAGB, laparoscopic adjustable gastric banding, SG, sleeve gastrectomy, RYGB, Roux-en-Y gastric bypass*.

**Figure 2:**
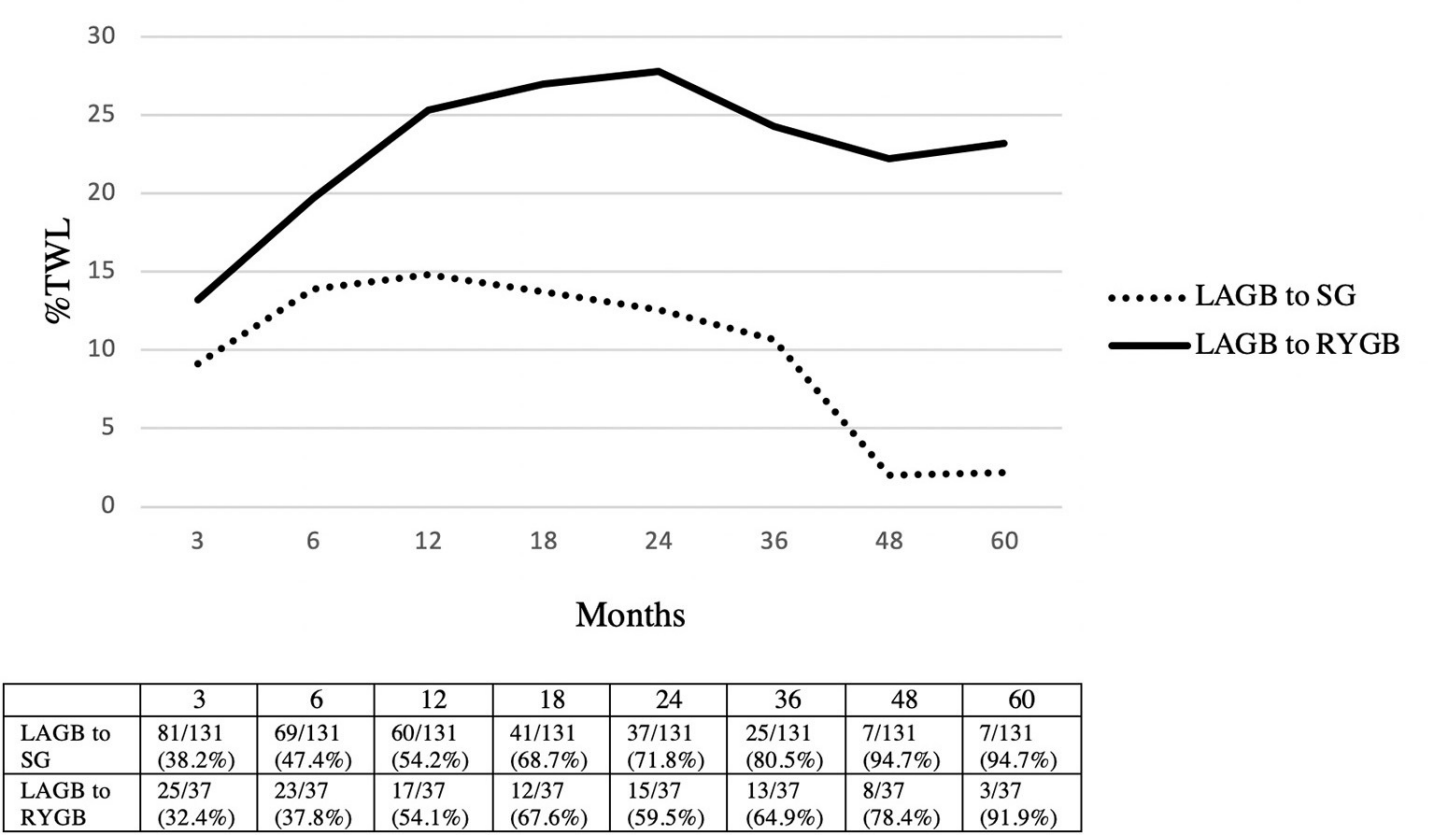
Weight loss outcomes in LAGB to SG vs. LAGB to RYGB. Patients seen/available patients (% loss to follow-up). LAGB, laparoscopic adjustable gastric banding; SG, sleeve gastrectomy; RYGB, Roux-en-Y gastric bypass; %TWL, total weight loss.

The mean follow-up was 20.3 (1–91) months in the LAGB to SG group and 25.2 (1–72) months in the LAGB to RYGB group. Greater %TWL was achieved in patients who underwent LAGB to RYGB conversions at 3 months (SG: 9.1% vs. RYGB: 13.2%, *p* = 0.006), 6 months (SG: 13.9% vs. RYGB: 19.7%, *p* = 0.002), 12 months (SG: 14.8% vs. RYGB: 25.3%, *p* < 0.001), 18 months (SG: 13.7% vs. RYGB: 27%, *p* = 0.001), 24 months (SG: 12.6% vs. RYGB: 27.8%, *p* < 0.001), 36 months (SG: 10.7% vs. RYGB: 24.3%, *p* = 0.005), 48 months (SG: 2% vs. RYGB: 22.2%, *p* = 0.006), and 60 months (SG: 2.2% vs. RYGB: 23.2%, *p* = 0.004) (Figure 2).

## Discussion

RBS are increasing in incidence, with 5–8% of primary bariatric procedures requiring a revisional operation (19). Despite the higher complication rate of RBS, its effectiveness in achieving weight loss and improvement in obesity-related comorbidities has been demonstrated (20, 21). According to literature, LAGB has the highest revision rate (40–50%), while RYGB and BPD/DS have the lowest (10–20% and 5%, respectively) (22). Similarly, we found that LAGB (78.7%) was the most revised primary procedure. Moreover, RYGB (2.7%) and BPD/DS (0.4%) revisions were infrequent. As previously described by several authors, we also found that inadequate weight loss and weight regain were the most common indications for RBS, followed by GERD (19–22).

The influence of the surgical approach on RBS postoperative outcomes is a matter of debate (7–11, 19, 22). The laparoscopic approach has been shown to have fewer postoperative complications and decreased hospital LOS when compared to the conventional open approach (19). Compared to standard laparoscopy, the robotic approach may provide greater benefit in more complex procedures such as RBS. In these cases, precise dissection and suturing are required due to adhesions, altered surgical planes, and decreased vascularization (8, 23). This potential benefit has been demonstrated in primary bariatric procedures, where the robotic platform was associated with lower stricture rates in RYGB (24). However, this outcome might be influenced by the anastomosis technique used. A case-matched study compared perioperative outcomes of laparoscopic and robotic SG and RYGB revisions using the 2015–2017 Metabolic and Bariatric Surgery Accreditation and Quality Improvement Program (MBSAQIP) database (11). Overall morbidity rates were higher in robotic SG revisions (robotic SG: 6.7% vs. laparoscopic SG: 4.5%, *p* < 0.01) and lower in robotic RYGB revisions (robotic RYGB: 9.3% vs. laparoscopic RYGB: 11.6%, *p* = 0.02) when compared to the laparoscopic approach. There was also a higher reoperation rate in robotic revisional SG (robotic SG: 2.4% vs. laparoscopic SG: 1.5%, *p* = 0.02), but this was not seen in robotic revisional RYGB (robotic RYGB: 3.8% vs. laparoscopic SG: 3.9%, *p* = 0.96). Similar conversion rates (robotic SG: 0.3% vs. laparoscopic SG: 0.1%, *p* = 0.24; robotic RYGB: 0.7% vs. laparoscopic RYGB: 0.6%, *p* = 0.76) and mortality (robotic SG: 0% vs. laparoscopic SG: 0.1%, *p* = 0.28; robotic RYGB: 0.1% vs. laparoscopic RYGB: 0.2%, *p* = 0.40) were found between groups. However, this data should be interpreted with caution due to potential selection bias (unknown index procedures), and coding errors (11). In our experience utilizing laparoscopic and robotic approaches, overall morbidity was 11.3%, major morbidity 3.2%, and mortality 0.4%. Our conversion rate of 0.9% and reoperation rate of 3.2% are within the range of data reported in the literature. The low conversion rate indicates that revisional procedures can be safely performed in a minimally invasive fashion by experienced surgeons. Moreover, we found similar postoperative outcomes in both laparoscopic and robotic revisional cases.

RBS could also be needed in patients with severe malnutrition or refractory dumping syndrome. If conservatory measures fail, common channel prolongation or reversal to normal anatomy might be required. In the present series, two patients (one with a BPD/DS and one with a RYGB) underwent reversal for malnutrition and another (RYGB) for severe dumping syndrome. All of them had an uneventful postoperative recovery and their nutritional status significantly improved.

Despite the initial popularity of LAGB, longer-term studies have shown an increasing need for revisional surgeries (up to 52%) (14). There is no consensus about what constitutes the gold standard revisional procedure after LAGB. LAGB removal alone is associated with persistence or recurrence of obesity (25). SG and RYGB are the most commonly performed RBS with improved weight loss (4). Concerns about revisional SG include difficult fundus resection from adhesions and fibrous capsule surrounding the band, as well as the idea that a failed restrictive procedure should not be replaced by another restrictive procedure (26). However, comparative studies are limited with varying conclusions. Khan and colleagues found higher rates of readmission and/or reintervention in conversions to RYGB (SG: 0% vs. RYGB: 14.2%, *p* = 0.04) but comparable weight loss (15). Similarly, Janik et al. found increased bleeding (SG: 0.44% vs. RYGB: 2.66%, *p* < 0.001), anastomotic leak (SG:1.18% vs. RYGB: 2.07%, *p* = 0.07), 30-day readmission (SG: 3.69% vs. RYGB: 7.46%, *p* < 0.001), and 30-day reoperation rates (SG: 1.26% vs. RYGB: 3.25%, *p* < 0.001) in patients who underwent laparoscopic conversion to RYGB (17). On the contrary, we previously reported similar complication rates and weight loss outcomes in LAGB conversions to SG and robotic RYGB (16). Currently, with a larger sample size and longer follow-up period, we found similar short (SG: 3.8% vs. RYGB: 10.8%, *p* = 0.11) and long-term (SG: 0.8% vs. RYGB: 5.4%, *p* = 0.12) complication rates, but greater %TWL at 1, 2, and 3 years in patients converted to RYGB. The differences in safety and weight loss outcomes among studies might be due to variances in operative technique, surgical approach, experience, and loss to follow-up (which varied considerably among studies).

The primary limitation of this study is its retrospective nature. The comparative analysis of band to SG vs. band to RYGB conversions might be influenced by the surgical approach, as the majority of LAGB to SG conversions were performed laparoscopically (93.1%), while most of LAGB to RYGB conversions were performed robotically (91.9%). Another limitation is the high rate of loss to follow-up (51.4% at 1 year, and 68.2% at 2 years), which may affect weight loss outcomes. However, this study represents one of the largest single center experiences in minimally invasive RBS. Future randomized controlled trials are warranted to draw more definitive recommendations.

## Conclusion

Minimally invasive RBS can be safely performed in a wide spectrum of patients, indications, and index procedures with low complications rates. LAGB is the most commonly revised procedure due to weight loss failure and late complications. LAGB conversion to RYGB was associated with better weight loss outcomes compared to conversion to SG. However, future randomized controlled trials are warranted to draw more definitive conclusions.

## Data Availability Statement

The raw data supporting the conclusions of this article will be made available by the authors, without undue reservation.

## Ethics Statement

The studies involving human participants were reviewed and approved by IRB UIC. The patients/participants provided their written informed consent to participate in this study.

## Author Contributions

JX, ND, FS, AC, AG, and MM: conception and design of the study. All authors: acquisition, analysis, interpretation of data, and manuscript preparation and review. All authors contributed to the article and approved the submitted version.

## Conflict of Interest

FB has an education agreement with intuitive. The remaining authors declare that the research was conducted in the absence of any commercial or financial relationships that could be construed as a potential conflict of interest.

## Publisher's Note

All claims expressed in this article are solely those of the authors and do not necessarily represent those of their affiliated organizations, or those of the publisher, the editors and the reviewers. Any product that may be evaluated in this article, or claim that may be made by its manufacturer, is not guaranteed or endorsed by the publisher.
